# Robotic Assisted Laparoscopic Prostatectomy Performed after Previous Suprapubic Prostatectomy

**DOI:** 10.1155/2016/4573819

**Published:** 2016-11-02

**Authors:** Johnson F. Tsui, Michael Feuerstein, Seyed Behzad Jazayeri, David B. Samadi

**Affiliations:** Department of Urology, Lenox Hill Hospital, Hofstra Northwell School of Medicine, New York, NY, USA

## Abstract

Operative management of prostate cancer in a patient who has undergone previous open suprapubic simple prostatectomy poses a unique surgical challenge. Herein, we describe a case of intermediate risk prostate cancer in a man who had undergone simple prostatectomy ten years prior to presentation. The patient was found to have Gleason 7 prostate cancer on MRI fusion biopsy of the prostate for elevated PSA and underwent an uncomplicated robot assisted laparoscopic radical prostatectomy.

## 1. Introduction

Benign prostatic hyperplasia (BPH) is the most common disease in men overall. In the prostate specific antigen (PSA) era, it is likely that the practitioner will encounter patients with prostate cancer who have undergone previous prostate treatments. Although medical treatment of BPH with alpha blockers and 5-alpha reductase inhibitors may ameliorate the urinary symptoms, surgical intervention may be inevitable in some patients. Either transurethral resection of prostate (TURP) or simple prostatectomy can help in reducing the urinary symptoms associated with BPH. Accidental findings of cancerous tissue in TURP have been reported before and studies have described various treatment methods for this group of patients. However, to our knowledge, this is the first report of a patient treated with robotic assisted radical prostatectomy after experiencing prostate cancer ten years after simple prostatectomy.

## 2. Case Presentation

**Figure 1:**
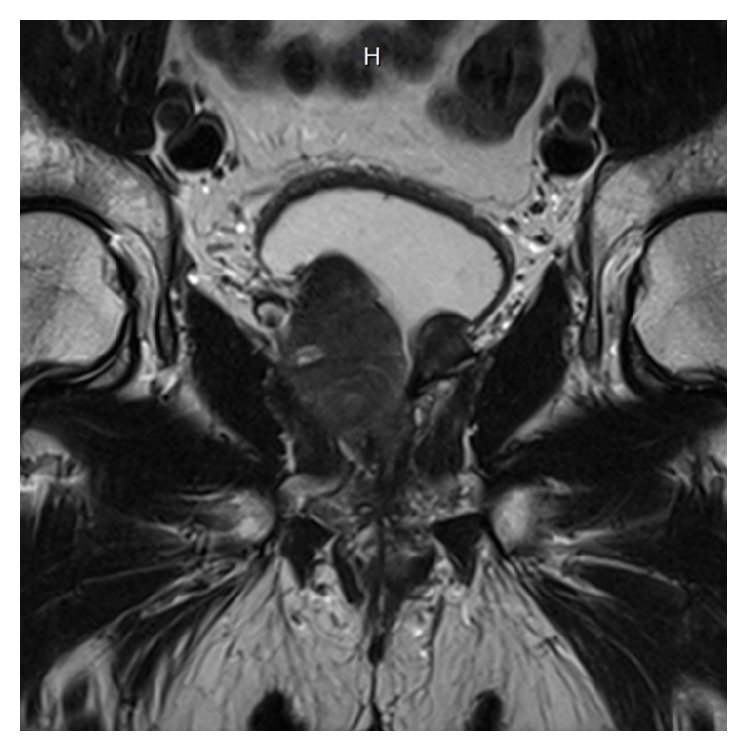
Coronal image of prostate showing extensive prostate cancer growth on the left side filling simple prostatectomy defect.

**Figure 2:**
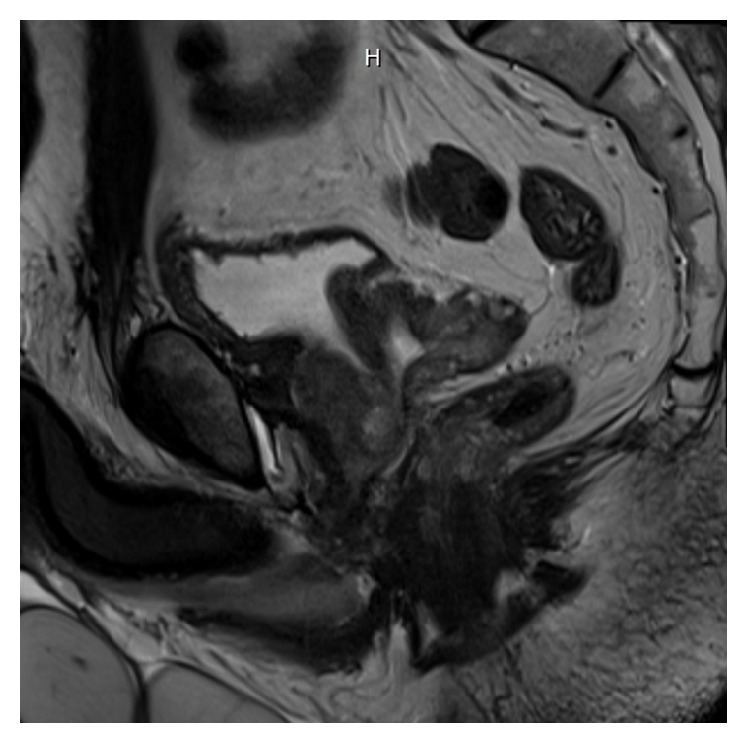
Axial image of the left side of the prostate from sagittal view demonstrating filling of simple prostatectomy defect with neoplasm.

A 69-year-old gentleman presented for consultation after being diagnosed with prostate cancer. His past medical history was significant for hypertension, hyperlipidemia, stable 5 mm left pulmonary nodule, mitral valve prolapse with moderate regurgitation, and BPH for which he underwent a simple prostatectomy ten years earlier. The patient elected decision to undergo simple prostatectomy a decade ago for BPH after alpha-blocker therapy and a short course of finasteride were ineffective in resolving his urinary retention. On this most recent consultation, physical exam was notable only for an estimated 60 g prostate with no nodules appreciated, suggesting cT1c prostate cancer. MRI showed extension of neoplastic tissue into the prostatectomy field (Figures 1 and 2). MRI fusion prostate biopsy performed for elevated PSA of 5.7 ng/mL demonstrated 4/12 positive cores, 2 Gleason 4+3, and 2 Gleason 3+4. Surgical history was also significant for an open left inguinal hernia repair that took place seven years ago. Preoperative imaging with CT of the abdomen and pelvis and whole body bone scan did not demonstrate any evidence of metastatic disease.

The patient elected to undergo robot assisted laparoscopic prostatectomy (RALP). Cystoscopy was performed prior to the procedure to assess bladder anatomy. At the start of the RALP procedure, the bladder was adhered anteriorly, requiring extensive lysis of adhesions and gently dissecting the bladder off the anterior abdominal wall. Upon entry into the space of Retzius, the endopelvic fascia was not incised to maximize nerve-sparing technique as part of Samadi Modified Advanced Robotic Technique (SMART) [1]. When the bladder neck was opened, ureteral stents were positioned by passing a wire through the side trocar and into the ureteral orifice using the robot and then passing the stent over the wire and through the port [2]. Stents were inserted in order to identify the exact location of the ureteral orifices, considering the change in normal anatomy following the previous surgery. Due to the change in the anatomy of the prostate and the patient's thin posterior bladder neck, tissue recognition was vital in this case. Posterior dissection was performed using cold scissors to minimize rectal damage and the bladder neck was opened. Nerve-sparing procedures were conducted using athermal technique by blunt dissection with round-tip scissors and performed in an interfascial plane, as opposed to an intrafascial one. The dorsal vein complex was cut with cold scissors just before removal of the specimen and then suture-ligated. The bladder neck was then reconstructed in a posterior tennis-racquet fashion with a narrower diameter of 18 Fr. After completion of reanastamosis, bilateral pelvic lymph node dissection was performed with minimal difficulty by removing the lymph package anterior to the obturator nerve and inferior to the external iliac vein. Total operative time was 145 minutes.

Pathologic examination of the specimens revealed a 76 g prostate with bilateral, Gleason 4+3 pT3a disease with extraprostatic extension, involving 58% of examined slides. The right posterior margin was positive focally. Lymphovascular invasion and peri- and intraneural invasion were present. No lymph nodes (0/3) were positive for disease.

Voiding cystogram performed 10 days post-op did not demonstrate any extravasation or abnormal findings. On the patient's most recent follow-up, approximately 9 weeks post-op, he was noted to be continent, with minimal difficulty voiding, and had a PSA of <0.01 ng/mL.

## 3. Discussion

To the best of our knowledge, there have been no previously reported cases of patients undergoing a RALP after previous suprapubic simple prostatectomy. Despite advances in management options for men with symptomatic BPH, open simple prostatectomy is still considered a practical option for patients with large glands, especially, when taking into account the volume of adenoma removed and long-term functional outcomes [3, 4]. Open prostatectomy for symptomatic BPH is reported to comprise about 3% of all prostatectomies performed in the United States [5].

Among patients who have undergone previous simple prostatectomy, the incidence of prostate cancer is not well described. Nevertheless, the presence of prostate cancer in remaining prostate tissue after a simple prostatectomy is not unimaginable, as incidental prostate cancer discovered in tissue obtained from TURP has been described.

Historically, prostate cancer has been identified in TURP specimens without prior diagnosis in 5 to 13% of patients [6]. A recent study by Sakamoto et al. found incidental prostate cancer in 10% of patients who underwent TURP, with 4% of the total cohort having stage T1b and/or Gleason ≥7 [6]. In a larger cohort studied by Otto et al., 11/771 patients had incidental prostate cancer, 10 of whom had Gleason grade 3+3 disease and only one with Gleason grade 3+4 disease. Of these 11 patients, 7 were managed with active surveillance, 1 was managed with external beam radiation, and 3 were managed with radical prostatectomy [7]. These lower detection rates are consistent with the overall decrease in incidental prostate cancer in the PSA era [8].

Outcomes in patients who undergo RALP after TURP vary. Menard et al., in a cohort selected from the period of 1998 to 2005, compared men who have undergone RALP after TURP to those who have not previously had TURP. This study demonstrated similarities with 5-year biochemical recurrences and 2-year continence rates, with higher risk of anastomotic stricture and impotence in men who have had previous TURP [9]. The difference in potency between the two groups was attributed to difficulty in preserving the neurovascular bundle in those who have had previous TURP. Furthermore, a study conducted by Gupta et al. found that patients undergoing RALP after previous TURP have higher positive margin, biochemical recurrence, and incontinence rates than patients undergoing RALP without previous TURP [10]. More recently, Zugor et al. reported similar positive surgical margin rates, continence, and potency at 12 months but did not note prolonged operative time and time interval before return of continence and potency when comparing patients undergoing RALP who have had previous TURP to those that have not [11].

It is difficult to determine if the results described in patients undergoing RALP after TURP can be extrapolated to our specific case due to the higher degree of technical difficulty posed by our patient. Although he remains continent at 2-month follow-up, it is still early to assess potency and longer follow-up is required as erectile function can continue to recover even after 12 months postoperatively [12]. Herein, we describe such a case and it is our recommendation that, due to the unique surgical difficulties inherent in such a patient, resulting from changes in anatomy that interfere with identification of dissection planes, it is necessary that surgical management be performed by a highly experienced surgeon with extreme patience and precision, in effort to minimize possible injuries and complications [1].
